# Cysteine protease and cystatin expression and activity during soybean nodule development and senescence

**DOI:** 10.1186/s12870-014-0294-3

**Published:** 2014-11-18

**Authors:** Stefan George van Wyk, Magdeleen Du Plessis, Christoper Ashley Cullis, Karl Josef Kunert, Barend Juan Vorster

**Affiliations:** Department of Plant Production and Soil Science, Forestry and Agricultural Biotechnology Institute, University of Pretoria, Pretoria, 0002 South Africa; Department of Biology, Case Western Reserve University Cleveland, Cleveland, OH 44106 USA; Department of Plant Science, Forestry and Agricultural Biotechnology Institute, University of Pretoria, Pretoria, 0002 South Africa

**Keywords:** Cystatin(s), Cysteine protease(s), Programmed cell death, RNASeq, Senescence, Soybean, Symbiotic nitrogen fixation, Transcriptome

## Abstract

**Background:**

Nodules play an important role in fixing atmospheric nitrogen for soybean growth. Premature senescence of nodules can negatively impact on nitrogen availability for plant growth and, as such, we need a better understanding of nodule development and senescence. Cysteine proteases are known to play a role in nodule senescence, but knowledge is still fragmented regarding the function their inhibitors (cystatins) during the development and senescence of soybean nodules. This study provides the first data with regard to cystatin expression during nodule development combined with biochemical characterization of their inhibition strength.

**Results:**

Seventy nine non-redundant cysteine protease gene sequences with homology to papain, belonging to different subfamilies, and several legumain-like cysteine proteases (vacuole processing enzymes) were identified from the soybean genome assembly with eighteen of these cysteine proteases actively transcribed during nodule development and senescence. In addition, nineteen non-redundant cystatins similar to oryzacystatin-I and belonging to cystatin subgroups A and C were identified from the soybean genome assembly with seven actively transcribed in nodules. Most cystatins had preferential affinity to cathepsin L-like cysteine proteases. Transcription of cystatins Glyma05g28250, Glyma15g12211, Glyma15g36180 particularly increased during onset of senescence, possibly regulating proteolysis when nodules senesce and undergo programmed cell death. Both actively transcribed and non-actively transcribed nodule cystatins inhibited cathepsin-L- and B-like activities in different age nodules and they also inhibited papain and cathepsin-L activity when expressed and purified from bacterial cells.

**Conclusions:**

Overlap in activities and specificities of actively and non-actively transcribed cystatins raises the question if non-transcribed cystatins provide a reservoir for response to particular environments. This data might be applicable to the development of strategies to extend the active life span of nodules or prevent environmentally induced senescence.

**Electronic supplementary material:**

The online version of this article (doi:10.1186/s12870-014-0294-3) contains supplementary material, which is available to authorized users.

## Background

In plants, cystatins are natural and specific inhibitors of cysteine proteases of the papain C1A family that generally block C1A proteases by a tight and reversible interaction [1]. Several cystatin functions have been proposed, but all involve a balanced interplay with a cysteine protease to regulate proteolytic activity [2,3]. Research has so far provided strong evidence that plant cystatins regulate endogenous protein turnover during growth and developmental processes, including senescence and programmed cell death, and are further involved in accumulation and mobilization of storage proteins. A further key function is protection against plant pests where cystatins prevent cysteine protease activity required for protein digestion in pests [3,4].

Cysteine protease expression during nodule senescence has been previously reported [5-8]. Proteolytic activity in infected nodules limits the bacterial symbiosis and nitrogen fixation, with cytosolic leghemoglobin and the bacteriod as targets. In *Medicago trunctula* anti-sense inhibition of the cysteine protease CYP15A caused a delay in nodule senescence [9] and nodule lifespan was prolonged, when a nodule-specific papain-like cysteine protease (AsNODF32) was silenced [10]. However, despite strong evidence for cysteine protease involvement in nodule development and senescence, only limited detailed information is currently available on any specific cystatin function and activity in these development and senescence processes [6,8,11,12]. The most detailed analysis of participation of an endogenous cystatin in interaction with an endogenous cysteine protease during senescence has been the coordinated expression of the mRNAs of a cysteine protease and a cystatin in senescent spinach leaves where a senescence-related cysteine protease–cystatin complex was identified [13]. Further evidence of the *in vivo* regulation of cysteine protease have been provided by Pillay *et al.* [14] showing that co-expression of the rice cystatin OCI in tobacco plants protected recombinant proteins from degradation by lowering overall cysteine protease activity.

The Phytozome database (www.phytozome.net) currently contains over 300 cystatin-like sequences from the Viridiplantae kingdom, 706 C1 cysteine protease sequences and 362 C13 cysteine protease (VPE-type) sequences. The recent release of the complete soybean genome [15] as well as the release of a RNAseq atlas of genes expressed in fourteen different soybean tissues including nodules [16] has further allowed identification and characterization of all 19 soybean cystatins, irrespective of transcriptional activity, and 18 active cysteine proteases. Accurate studies are now possible to determine the cystatin and cysteine protease classes expressed in nodules and also to investigate if endogenous cystatins preferentially interact with specific target cysteine proteases in nodules. Our study was therefore aimed to provide a first insight into such interactions by identifying and characterizing all members of the cystatin and cysteine protease gene families in soybean nodules. We included both actively and non-actively transcribed cystatins and cysteine proteases identified through homology searches in the soybean genomic database. The nodule transcription profiles were developed with the technique of RNAseq [17] which allowed us to determine the expression of all oryzacystatin I-like cystatins, papain-like cysteine proteases, as well as vacuole VPE-type cysteine proteases in determinate soybean crown nodules during nodule development and senescence. Such VPE cysteine proteases resemble mammalian caspases and they contribute to the senescence process and PCD (Programmed Cell Death) [18], but might further activate pre-proteases by post-translational modification [19].

In our characterization, we were also interested to determine to which families and functional groups nodule cystatins and cysteine proteases belong as well as the cystatin substrate preference by testing *in vitro* produced cystatin proteins with various cysteine protease-containing extracts. Cystatins are part of subfamily B of the I25 cystatin family and in cereals they can be divided into various functional groups (A, B and C) with most cystatins belonging to groups A and C [20]. Group A cystatins, which efficiently inhibit cathepsin L-like cysteine-proteases, are preferentially expressed in dry and germinating seeds whereas group C1 cystatins, which are potent inhibitors of C1A peptidases, are mostly expressed in developing seed endosperms. Cysteine proteases cluster into different subfamilies [21] with cysteine proteases closest to papain clustering with subfamily XCP1 represented by the *Arabidopsis thaliana* genes At1g20850 and At4g35350. Cysteine proteases with cathepsin-L-like activity can closely cluster with subfamily RD21 consisting of RD21A (*A. thaliana* gene At1g47128), RD21B (At5g43060) and RD21C (At3g19390). A C-terminal granulin domain is characteristic of the RD21 subfamily. Cysteine proteases with cathepsin-L-like activity can further cluster with the SAG12 subfamily. Cysteine proteases with cathepsin-F-like activity cluster with subfamily RD19 with members RD19A (At4g39090), RD19B (At2g21430) and RD19C (At4g16190) and RD19 members have a characteristic ERFNAQ motif in the pro-domain. Cysteine proteases with cathepsin-H-like activity cluster with members of the AALP (At5g60360) and ALP2 (At3g45310) subfamily.

We were finally also interested to determine the interaction affinities between selected actively and non-actively transcribed cystatins during nodule development and senescence. This should provide information about the relative activities and specificities of both expressed and non-expressed cystatin genes in soybean. In our study, we found an overlap in the activities and specificities of the expressed and non-expressed cystatin genes raising the question of whether the non-transcribed cystatins provide a reservoir for responses to particular environments.

## Results

### Cystatin identification

**Figure 1:**
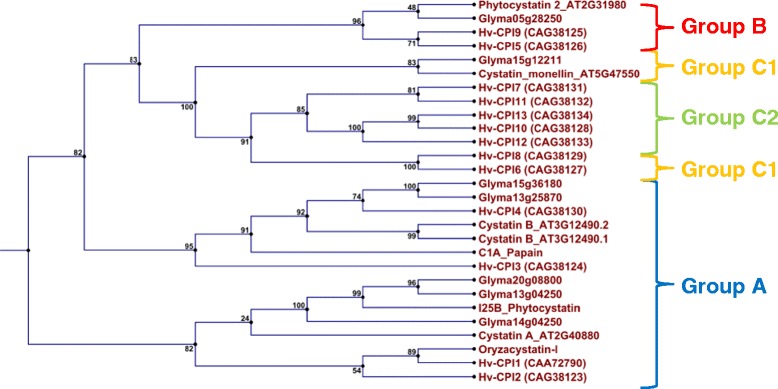
**Mapping of transcribed soybean nodule cystatins to different I25 cystatin subfamilies.**

All expressed nodule cystatins were identified from our RNAseq data. When the oryzacystatin-I (conserved region 1EQK_A) was used for comparison as a model cystatin, 25 cystatin sequences were identified in the assembled genome; of these 20 were non-redundant sequences (Additional file 1). When we carried out a phylogenetic genetic analysis of cystatins by comparison with cystatins from different I25 cystatin subfamilies (Figure 1), Glyma13g04250 and Glyma20g08800, transcribed in nodules during nodule development and senescence, had high similarity to group A cystatins (*Vigna unguiculata* cystatin, OCI, HvCPI-1 and HvCPI-2) [20]. Glyma13g04250 was further paralogous to Glyma14g04250 with identical location, but on a different chromosome. Also, the two cystatins Glyma13g25870 and Glyma15g36180 were highly similar to Cystatin B (At3g12490) and HvCPI-4 (group A) and Glyma05g28250 was further highly similar to group B cystatins (cystatin 2 (At2g31980), HvCPI-5 and HvCPI-9). They also contained a C-terminal extension with a SNSL amino acid motif enabling them to inhibit legumain C13 cysteine proteases [22]. Finally, Glyma15g12211, which was the most abundant cystatin in nodules, was similar to group C (subgroup C1) cystatins (Monellin cystatin (At5g47550), HvCPI-6 and HvCPI-8).

We also searched all cystatin sequences for signal peptides indicating their possible cellular localisation (Additional file 2). Glyma05g28250, Glyma07g39590 and Glyma13g25870 might be localised in the secretory pathway, whereas Glyma13g04250, Glyma14g04250 and Glyma20g08800 are localised to any location, except the chloroplast, mitochondrion or secretory pathway. Localisation of Glyma15g36180 was not reliable and the cystatin could be located in either the mitochondrion or the secretory pathway.

### Cysteine protease identification

**Figure 2:**
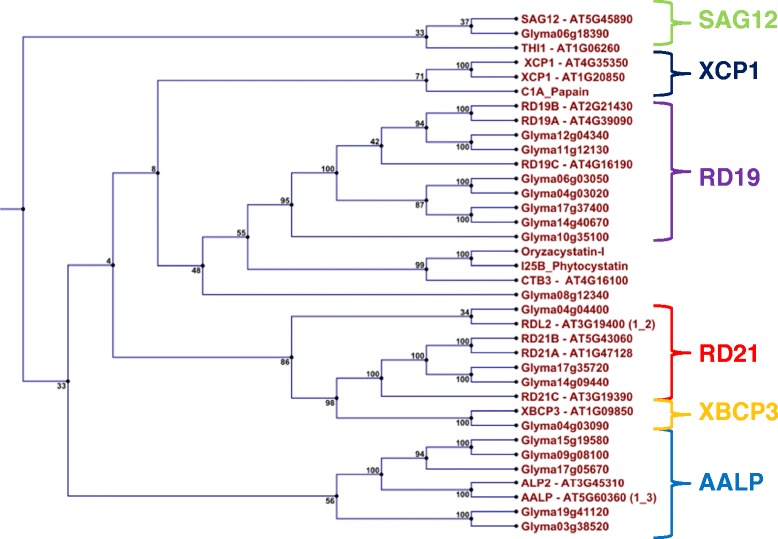
**Mapping of transcribed cysteine proteases to sub-families and functional groups with similarity to the C1 cysteine protease papain.**

A total of 99 cysteine protease sequences with homology (1E ≤ −1.0) to the model cysteine protease papain (E.C.3.4.22.2) were further identified from the soybean genome assembly (Additional file 3). Several sequences were alleles, paralogos and orthologos of other cysteine protease gene sequences. From these we identified 79 non-redundant cysteine protease gene sequences which had similarity to members of eight different cysteine protease sub-families. Seven sub-families were distinguished from our expression data and we identified confidently five functional groups (Figure 2). However, none of the identified soybean cysteine proteases clustered with papain (subfamily XCP1). Cysteine proteases with cathepsin-L-like activity included Glyma04g03090 (closely clustering with subfamily RD21), as well as the two proteases Glyma14g09440 and Glyma17g35720 (similar to subfamily RD21 members). We also confirmed the C-terminal granulin domain, characteristic of the RD21 subfamily, in these cysteine proteases. Glyma04g04400 (cathepsin-L-like activity) had highest similarity to RDL2 (Arabidopsis gene At3g19400) and closely clustered with the RD21 subfamily members. Finally, Glyma04g36470 and Glyma06g18390 (cathepsin-L-like activity) were highly similar to members of the SAG12 subfamily despite absence of the additional C amino acid in the CG*C*CWAFS motif.

Seven proteases with cathepsin-F-like activity (Glyma04g03020, Glyma06g03050, Glyma10g35100, Glyma11g12130, Glyma12g04340, Glyma14g40670, Glyma17g37400) were highly similar to subfamily RD19 members. However, the ERFNAQ motif (instead of the ERFNIN motif in the pro-domain) characteristic of the RD19 subfamily, was absent. Glyma08g12340, which had no significant similarity to any specific subfamily, was closest to the two subfamilies RD19 or CTB3. Further cysteine proteases with cathepsin-H-like activity included Glyma09g08100, Glyma15g19580 and Glyma17g05670, which had high similarity to AALP and ALP2. The three proteases also had an N-terminal NPIR vacuolar targeting signal and other RD21 subfamily motifs (except that the ATC motif was lacking in Glyma09g08100). Although Glyma03g38520 and Glyma19g41120 had similarity to this subfamily, they contained an ECGIE motif in the C terminus, characteristic of subfamily CTB3.

### Cystatin transcription

**Figure 3:**
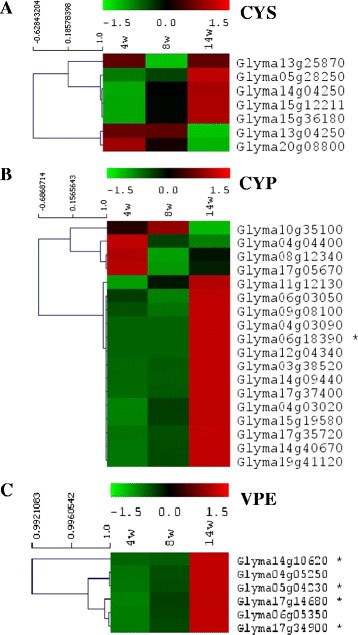
**Expression changes of cystatins, cysteine proteases and vacuolar processing enzymes. (A)** Expression of cystatins (**CYS**) **(B)** cysteine proteases (**CYP**) and **(C)** vacuolar processing enzymes (**VPE**) in 4, 8 and 14 week old nodules expressed as FPKM (transcript abundances in fragments per kilobase of exon per million fragments mapped). Colour scale represents transcription for each time point normalized by subtracting the mean/median of three values from each individual value for each gene reduced by SD/RMS. * indicates significant change (p < 0.05) in transcription between individual time points. Multi-experiment viewer (MeV v4.8.1) software package was applied to graphically represent data [52].

We then investigated the nodule cystatin and cysteine protease transcriptome at various time points (4, 8 and 14 weeks) of soybean nodule development and senescence (Figure 3). The time point at 4 weeks represents initial nodule development, 8 weeks mature nodules actively fixing nitrogen, and 14 weeks senescing nodules. After three biological replicates were produced for each time point and pooled, RNA was sequenced producing a total of ~40 million paired reads for each time point. A cystatin, or cysteine protease, was considered transcriptionally active if a FPKM ≥5.0 was obtained in any of the three time points [23]. If a cystatin, or cysteine protease, was not transcriptionally active (FPKM <5) at all 3 of the time points, the cystatin, or cysteine protease, was considered transcriptionally inactive.

**Figure 4:**
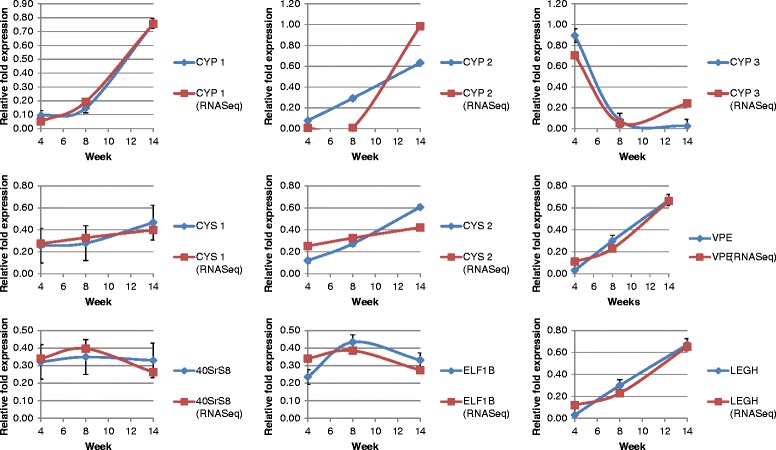
**Relative expression measured by quantitative real-time PCR of soybean cysteine proteases, cystatins, leghemoglobin and a VPE at each time point (4, 8 and 14 weeks) and corresponding FPKM abundance estimates derived from RNA-Seq mapping.**

We first compared our FPKM data with previous published data available online at SoySeq database (http://soybase.org/soyseq/) on the SoyBase website [16] where various tissue types have been analysed 20–25 days after inoculation (comparable to our 4 weeks data). Transcript abundance estimates from the two studies were directly comparable (data not shown). From a total of 20 putative soybean cystatins identified with the model I25B cystatin OC-I, only seven cystatins were transcriptionally active in nodules (Figure 3A). Glyma13g04250 and Glyma20g08800 had highest expression after 4 weeks but their expression decreased when nodules aged (Figure 3A). In contrast, transcription of Glyma05g28250, Glyma15g12211 (the most abundant cystatin) and Glyma15g36180 increased in the later stages of nodule development (Figure 3A), although none of these cystatins had statistically significant (p ≤0.05) transcriptional changes. The two remaining cystatins, Glyma13g25870 and Glyma14g04250, did either not change (Glyma13g25870) or expression was below, or close to, the detectable threshold level (Glyma14g04250). We also validated our RNAseq data by quantitative real-time PCR where tested transcripts were selected on the basis of being representative for each investigated gene family. Determination of relative fold-expression of transcripts during development confirmed our RNAseq data indicating the fidelity of our RNAseq analysis approach (Figure 4).

### Cysteine protease transcription

From the initial 99 putative cysteine protease sequences homologous to the model C1 cysteine protease papain, 18 cysteine proteases were transcriptionally active in nodules during at least one time point (Figure 3B). Glyma15g19580 (cathepsin-H like activity) was the most abundant cysteine protease in 4 weeks old nodules with Glyma17g37400 (cathepsin-F like activity) the most abundant at 14 weeks. Transcription of the majority of cysteine proteases increased with the onset of senescence, with five cysteine proteases (Glyma04g04400, Glyma08g12340, Glyma10g35100, Glyma11g12130 and Glyma17g05670) highly expressed in 4 and 8 weeks old nodules. None of the cysteine protease transcription changed significantly (p ≥0.05) except Glyma06g18390 transcription, with a very low relative abundance, which changed (p ≤0.05) due to senescence (Figure 3B).

We also investigated VPE protease (C13 cysteine proteases) transcription (Figure 3C). These proteases resemble mammalian caspases. VPE transcription significantly increased during nodule senescence and transcription of four sequences (Glyma05g04230, Glyma14g10620, Glyma17g14680, Glyma17g34900) significantly (p ≤0.05) increased (4.0 log_2_-fold change) for Glyma14g10620 and Glyma17g34900, with Glyma17g34900 having the largest increase in transcription due to senescence (Figure 3C). From the seven VPE gene sequences identified in the genome, only Glyma16g07190 was not transcribed during nodule development.

### Cystatin inhibition strength and specificity

In a next step, we carried out cysteine protease activity measurements with nodule extracts to determine potency of transcribed cystatins. Fluorometric interaction assays were used with either commercially available cathepsin-L or cathepsin-B as well as isolated nodule protein extracts representing the total proteolytic complement active in nodules. To establish a preferential binding for each cystatin, we first tested cystatin potency with commercially available enzyme preparations for cathepsin-L and cathepsin-B. Cystatins transcribed in nodules had generally stronger affinity for cathepsin-L than cathepsin-B, with Glyma13g27980 and Glyma14g04250 equally effective in preventing both cathepsin activities (Table 1). Further, Glyma15g36180 inhibited cathepsin-L, but was unable to inhibit cathepsin-B, even when an inhibitor concentration of 1 mM was used. In contrast, cystatins not transcriptionally active in nodules showed higher inhibition rates of cathepsin-L, with Glyma18g12240 inhibiting both cathepsin-L and -B. Glyma14g04260’s second domain and both domains of Glyma14g04291 were further unable to inhibit cathepsin-B, even at a concentration of 1 mM (Table 1).

**Table 1:** **Inhibition (%) of protease activity by actively and non-actively transcribed cystatins during nodule life-span**

**Cystatin**	**Cathepsin L-like activity**				**Cathepsin B-like activity**			
	**(% inhibition)**				**(% inhibition)**			
	**4 weeks**	**8 weeks**	**14 weeks**	**p** ≤ **0.05**	**4 weeks**	**8 weeks**	**14 weeks**	**p** ≤ **0.05**
Positive control (E64)	50.3 ± 1.1	26.4 ± 5.0	31.9 ± 4.5	*ac	37.2 ± 2.3	NI	NI	*ac
OCI (1 μM)	47.4 ± 1.3	28.2 ± 2.3	22.7 ± 7.3	*ac	44.9 ± 3.8	NI	NI	*ac
*Actively transcribed*								
Glyma05g28250	36.1 ± 0.5	31.5 ± 0.9	30.6 ± 0.4	ns	32.8 ± 1.4	32.8 ± 1.4	NI	*bc
Glyma13g04250	26.4 ± 0.9	NI	29.7 ± 1.8	*ab	27.6 ± 2.3	27.6 ± 2.3	24.9 ± 3.2	*ab
Glyma13g27980	33.2 ± 2.3	NI	NI	*ac	42.0 ± 0.2	42.0 ± 0.2	NI	*ac
Glyma14g04250	NI	NI	21.9 ± 1.6	*bc	NI	NI	NI	ns
Glyma15g36180	49.9 ± 5.3	28.4 ± 3.1	NI	*abc	48.7 ± 4.5	48.7 ± 4.5	NI	*ac
Glyma20g08800	NI	NI	NI	ns	NI	NI	32.5 ± 3.2	*ab
*Non-actively transcribed*								
Glyma04g10360	38.6 ± 2.9	32.0 ± 3.9	39.0 ± 3.5	ns	35.3 ± 5.5	30.9 ± 5.5	28.6 ± 5.8	ns
Glyma07g39590	47.5 ± 3.2	39.1 ± 9.5	51.3 ± 5.1	*b	42.3 ± 5.3	26.9 ± 8.7	34.0 ± 2.9	*a
Glyma08g11210	43.6 ± 3.8	28.2 ± 1.8	33.5 ± 4.3	*abc	42.1 ± 4.4	NI	NI	*ac
Glyma14g04260	58.9 ± 1.1	37.8 ± 4.9	36.2 ± 3.3	*ac	46.4 ± 1.8	NI	NI	*ac
(1st domain)								
Glyma14g04260	36.6 ± 4.9	NI	NI	*ac	39.8 ± 5.6	NI	NI	*ac
(2nd domain)								
Glyma14g04291	42.1 ± 3.3	NI	NI	*ac	30.9 ± 5.6	NI	NI	*ac
(1st domain)								
Glyma14g04291	40.8 ± 8.3	NI	NI	*ac	28.6 ± 8.4	NI	NI	*ac
(2nd domain)								
Glama18g12240	54.0 ± 2.6	43.1 ± 1.9	51.5 ± 3.7	*a	36.6 ± 5.8	28.3 ± 3.9	22.4 ± 7.4	*c

*a (4 weeks – 8 weeks); *b (8 weeks – 14 weeks); *c (4 weeks −14 weeks); NI represents inhibition ≥20%; *significant at p ≤0.05. Blank values for Cathepsin L-like activity and Cathepsin B-like activity was 0.5 ± 0.7 FU/sec and 0.0 ± 0.3 FU/sec, respectively. The negative control values for Cathepsin L-like activity and Cathepsin B-like activity was 42.5 ± 1.6 FU/sec and 28.2 ± 0.8 FU/sec, respectively.

We then tested cystatin potency against various nodule extracts (Table 2). We first used the model rice cystatin OC-I as well as the cysteine protease inhibitor E64. OC-I and E64 both prevented cathepsin-L-like activity in 4 weeks old nodules but were less efficient against extracts derived from 8 and 14 weeks old nodules (Table 2). Both inhibitors also prevented cathepsin-B-like activity in an extract of 4 weeks old nodules. We then compared OC-I and E64 potency with the potency of various recombinant soybean cystatins either actively transcribed or non-active in nodules (Table 2). Cystatins tested were generally more active against extracts from younger nodules (Table 2). Five of the cystatins actively transcribed in nodules blocked cysteine protease activity in nodule extracts. However, only Glyma05g2850 inhibited cathepsin-L-like activity in nodule extracts from all three time points (4, 8, and 12 weeks) and cathepsin-B-like activity in extracts derived from 4 and 8 weeks old nodules. The most potent cystatin among the expressed cystatins was Glyma15g36180 and potency of this cystatin was comparable to OC-I and E64 when either cathepsin–L or B activity was measured in an extract derived from 4 weeks old nodules.

**Table 2:** **Expression and inhibitory potency of cystatins against proteases from different aged nodules**

**Cystatin**	**Expression**	**Cat-L inhibition**	**Cat-B inhibition**
**Active**			
*4 weeks*			
Glyma05g28250	+ (22.65)	+ (36.1%)	+ (32.8%)
Glyma13g04250	+ (97.58)	+ (26.4%)	+ (27.6%)
Glyma14g04250	(−) (2.14)	(−)	(−)
Glyma15g36180	+ (26.34)	++ (49.9%)	++ (48.7%)
Glyma20g08800	+ (85.83)	(−)	(−)
*14 weeks*			
Glyma05g28250	+ (39.78)	+ (30.6%)	(−)
Glyma13g04250	+ (63.86)	+ (29.7%)	(+) (24.9%)
Glyma14g04250	(+) (12.38)	(+) (21.9%)	(−)
Glyma15g36180	+ (55.64)	(−)	(−)
Glyma20g08800	+ (56.25)	(−)	(−)
**Non-active**			
*4 weeks*			
Glyma04g10360	(−) (0)	+ (38.6%)	+ (35.3%)
Glyma07g39590	(−) (2.09)	+ (47.5%)	+ (42.3%)
Glyma08g11210	(−) (0)	+ (43.6%)	+ (42.1%)
Glyma18g12240	(−) (0.28)	++ (54.0%)	+ (36.6%)
Glyma13g27980	(−) (0)	+ (33.2%)	+ (42.0%)
*14 weeks*			
Glyma04g10360	(−) (0)	+ (39.0%)	+ (28.6%)
Glyma07g39590	(−) (1.23)	++ (51.3%)	+ (34.0%)
Glyma08g11210	(−) (0)	+ (33.5%)	(−)
Glyma18g12240	(−) (0.58)	++ (51.5%)	(+) (22.4%)
Glyma13g27980	(−) (0)	(−)	(−)

++ strong, + medium. (+) low and (−) no cystatin expression/or activity (tested up to 1 mM). Expression indicated as measured FPKM abundances and activity indicated as% inhibition.

Finally, we were also interested in testing cystatins not actively transcribed during nodule development. These cystatins were generally more active against nodule extracts than cystatins actively transcribed in nodules (Table 2). All non-transcribed cystatins had potency comparable to OC-I and E64 when tested against an extract derived from 4 weeks old nodules. Among them, Glyma14g04260 domain 1 and Glyma18g12240 had highest inhibition of all tested cystatins with 58.9% and 54% inhibition respectively. Three cystatins (Glyma04g10360, Glyma07g39590 and Glyma18g12240) inhibited cathepsin-L as well as cathepsin-B like activity in extracts derived from all three time points. Glyma07g39590 was further the most potent of all tested cystatins against cathepsin-L and B activity in an extract derived from 14 weeks old senescent nodules.

## Discussion

We identified 8 cystatin genes expressed in soybean nodules using RNAseq. Since we carried out macro-dissection of crown nodule tissue, and not micro-dissection with selected tissues, RNAseq data represented transcription profiles of the entire nodule and also contained transcripts from areas surrounding the senescing nodule cortex. When we compared their transcription with already published RNAseq data from various other tissue types [16], none of the identified nodule cystatins was uniquely transcribed. Several cystatins were further actively transcribed during nodule development and senescence but not exclusively transcribed at a particular time point like senescence. Only Glyma05g28250 was actively transcribed, and also inhibited cathepsin-L-like activity in nodule extracts, at all three selected time points. The cystatin very likely plays a maintenance role and regulates cysteine protease activity throughout nodule development and senescence. The other actively transcribed cystatins were only capable of inhibiting specific types of cysteine proteases activity (cathepsin L or B) at specific time points. Cathepsin B is a member of the peptidase C1 family and this cysteine protease is required for PCD involved in the plant disease resistance hypersensitive response [24]. Transcription of cystatins Glyma05g28250, Glyma15g12211, Glyma15g36180 increased by about two-fold during the onset of senescence with concurrent co-induction of several cysteine proteases. These cystatins very likely regulate proteolysis when nodules senesce and undergo PCD to maintain nitrogen fixation in determinate soybean nodules for as long as possible.

Cystatins, which are part of subfamily B of the I25 cystatin family, belong to various groups (A, B and C) from to our phylogenetic analysis [20]. However, any classification solely based on phylogenetic analysis might not accurately provide information about a particular function [20]. Several nodule cystatins, almost equally transcribed during nodule development and senescence, had high similarity to group A cystatins. In cereals, group A cystatins, including rice cystatins, efficiently inhibit cathepsin L-like cysteine-proteases and they are preferentially expressed in dry and germinating cereal seeds. They possibly regulate endogenous enzymes involved in the mobilization of stored proteins upon germination [20,25,26].

The nodule group A cystatin cluster also contained two cystatins, Glyma13g25870 and Glyma15g36180, with a C-terminal extension. Such an extension was also found in Glyma05g28250, highly similar to group B cystatins. Plant cystatins with a carboxy-terminal extension contain a SNSL amino acid motif and inhibit cysteine proteases of the legumain C13 family (VPEs) [22]. Their consistent transcription during nodule development and increase of transcription found for Glyma15g36180 and Glyma05g28250 when nodules senesce, indicates that they are very likely produced to tightly control cell disruption and activation of any cysteine proteases that may compromise nitrogen fixation. VPE proteases resemble mammalian caspases and they contribute to the senescence process and PCD by contributing to the collapse of the vacuole membrane with release of proteases into the cell [18]. There is also evidence that VPEs play a regulatory role activating pre-proteases by post-translational modification, leading to maturation and proteolytic activity upon removal on the I19 inhibitory domain [19]. Cysteine proteases, expressed as pre-proteins, consist of an I29 inhibitor domain preventing non-specific activity [27]. In our study, transcription of the entire set of nodule VPE cysteine proteases strongly increased coinciding with the progression of senescence. VPEs are therefore predominantly transcribed in senescent nodules and might play an important role in the activation of cysteine proteases. These activated cysteine proteases finally degrade both the bacteroids and nodule cells [28-32] and correlates with nitrogenase activity decrease [8] as well as decrease in both crown nodule biomass and nodule number [12].

Glyma15g12211, identified in the Phytozome database, was the most abundant nodule cystatin with similarity to group C1 cystatins. This cystatin was almost four-times higher transcribed than all other actively transcribed cystatins in nodules. The Glyma15g12211 was identical to the previously described Glyma15g12210 [16] which was found to be highly transcribed both in nodules and in other tissues including seeds. In cereals, group C1 cystatins are preferentially expressed in seeds, particularly in developing endosperms, and are potent inhibitors of C1A peptidases [20]. Future research is needed to identify the specific target cysteine proteases and why Glyma15g12211 is preferentially expressed in nodules.

We also identified cystatins not actively transcribed in nodules. When expressed *in vitro*, these cystatins had a much broader range of inhibitory activity against the nodule proteolytic complement than actively transcribed cystatins. They had nearly double the inhibitory capacity towards cathepsin-L-like cysteine protease activity, and also partially towards cathepsin-B-like cysteine protease activity, compared to actively transcribed cystatins. This might indicate that proteolytic activity should not be compromised by extensive cystatin expression with the onset of senescence and remobilization of nitrogen. However, these non-actively-transcribed cystatins might also have other specific functions and are only activated under certain biotic and abiotic stress conditions to prevent premature nodule death.

Based on our RNAseq data, 18 cysteine proteases were actively transcribed in nodules during development and senescence. Identified cysteine proteases were further functionally diverse belonging to different proteolytic sub-families. Transcript abundance of cysteine proteases at early and mature nodule development was relatively constant, with different cysteine proteases contributing toward the overall proteolytic complement (cathepsin-B-, F-, L- and H-like). Most of our tested nodule cystatins had preferential affinity to cathepsin L-like cysteine proteases. With the onset of senescence, however, cysteine protease transcript abundance was nearly doubled and correlated with senescence progression. However, only transcription of Glyma06g18390, which was very lowly transcribed, changed significantly due to the onset of senescence. This cysteine protease is homologous to senescence-related SAG12 (At5g45890). However, in a previous comprehensive transcriptomics study in *Medicago truncatula* to understand *Medicago* nodule senescence, four cysteine protease genes highly homologous to SAG12 were the most abundant [33]. Future research has to determine the reason for such transcription difference of SAG12 homologous cysteine proteases in soybean determinate nodules and *Medicago* indeterminate nodules.

To analyze any endogenous cystatin function in nodules, it is crucial to identify their possible endogenous target cysteine proteases. Only little is so far known about any possible direct interaction between cystatins and their target proteases *in vivo* [4]. We therefore searched cystatin and cysteine proteases sequences for signal peptides to obtain some information about their cellular localisation. Cystatins Glyma05g28250, Glyma07g39590 and Glyma13g25870 are likely localised in the secretory pathway. The ER has a vast protein storage protein capacity and relatively low proteolytic activity and cystatins might contribute to low proteolytic activity in this organelle [34]. Nodule cystatins, such as Glyma05g28250, Glyma07g39590 and Glyma13g25870 with signal peptides, would be able to interact with C1A proteases that also have secretion sequences in their gene sequences [2]. MJ Chrispeels and NV Raikhel [35] further predicted that the open reading frame of two cysteine proteases contain a putative vacuolar targeting signal. We also found such signal for Glyma04g36470 and Glyma06g18390, despite that Glyma06g18390 was explicitly expressed during nodule senescence which raises the question of the particular Glyma06g18390 function when targeted to the vacuole. One possible explanation is that this vacuolar targeting signal directs these cysteine proteases to the bacteroid-containing symbiosome compartment for bacteroid protein degradation affecting peribacteriod membrane stability. Rupture of the peribacteriod membrane then eliminates the microbial partner [30].

## Conclusions

In our study the phylogenetic relationship and transcription of individual components of the cysteine protease–cystatin system in soybean nodules during nodule development and senescence were characterised. Several of these components, including legumains, had coordinated transcription during nodule senescence strongly indicating their direct involvement in nodule senescence. Our study has, overall, created new knowledge about types of cystatin and cysteine protease transcribed, timing of coordinated transcription of the two components of the system and inhibitory activity of actively transcribed and non-transcribed cystatins in nodules for more future detailed interaction studies. Application of this knowledge might ultimately allow development of engineering strategies to circumvent particularly stress-induced premature senescence in nodules [36]. The potential of using cystatins to regulate cysteine protease activity and thereby agronomical important traits (such as stress tolerance, delayed leaf senescence, etc.) was demonstrated in a transgenic soybean line overexpressing a cystatin [36]. Prolonging the period of active nitrogen fixation, due to delayed nodule senescence by regulating cysteine protease activity, might provide the benefit of better soybean growth and improving yield [10] and potentially contributing to future food security [37]. However, since still little is currently known about the *in vivo* interaction between the two components of the system, we are currently determining, as a next step, their interaction by *in vitro* assays with both purified recombinant cystatin and purified cysteine proteases and investigating their individual localisation by immuno-histochemistry.

## Methods

### Identification of cysteine proteases and cystatins in soybean

The Soybean Genome Database [http://soybase.org/], Phytozome Database [http://www.phytozome.net/soybean] and NCBI-BLAST [http://blast.ncbi.nlm.nih.gov/] online resources were searched to identify the entire family of cystatins and cysteine proteases and cystatins in *Glycine max*. For identification of cystatin homologues, oryzacystatin-I [PDB: 1EQK_A] from rice was applied as model representative of the I25 family of cysteine protease inhibitors and for identification of cysteine protease homologues in soybean, the cysteine protease papain [E.C.3.4.22.2; GenBank: P00784] from *Carica papaya* was used as model representative for the C1A cysteine protease family. BLASTn, tBLASTx and BLASTp programmes were applied to identify all I25 cystatins and all C1 cysteine proteases with an E-value cut-off of 1E-1.0 to identify homologous gene sequences. Since the database was first accessed during July and November of 2011, the gene nomenclature was maintained to correspond to the Glyma 1.89 reference assembly [15] which was applied for RNA-Seq read mapping. Gene sequences identified for investigation are listed in Additional files 1 and 3.

### Plant material and RNA preparation

Soybean (*Glycine max* L. Merr.) seeds of the commercial cultivar Prima 2000 were obtained from Pannar Seed in South Africa. Each pot was inoculated with 0.5 g of SoyGro inoculum (SoyGro Bio-Fertilizer Limited), containing *Bradyrhizobium japonicum* of the strain WB74-1, prior to planting in fine vermiculite (Mandoval PC). Plants were grown under controlled conditions, 13-h photoperiod at a light intensity of 600 mmol.m^−2^.s^−1^, with 3-h of supplementary light from metal-halide lamps and using a day/night temperature of 25°C/17°C and 60% relative humidity. Distilled water was used for plant watering and twice a week watered with a nitrogen-poor nutrient solution [38]. Watering regime promotes symbiotic relationship between the plant and the *Rhizobium* stimulating nodules with high symbiotic nitrogen fixation [39]. Crown nodules, harvested from a minimum of three plants at time points, 4, 8 and 14 weeks of development, were flash frozen in liquid nitrogen and stored at −80°C until RNA extraction. Three biological replicates were pooled for RNA extraction with a Qiagen RNeasy® kit (Qiagen, Germany). RNA quantity was measured with a Thermo Scientific NanoDrop 2000 with RNA quality analysed on a 2% agarose gel prior to sequencing at Case Western Medical Institute. Illumina mRNA-SEQ kit was applied for sample preparations and RNAseq libraries were generated with Illumina Genome AnalyzerIIκ.

### Transcriptome sequencing, data processing, normalization and data mining

Sequenced RNA was analysed with the Galaxy server [http://galaxy.bi.up.ac.za/] (Bioinformatics Unit, Forestry and Agricultural Biotechnology Institute, University of Pretoria). Glyma1.89 genomic assembly and transcriptome models, available on Phytozome [15], were applied as reference for annotation of mapping reads. RNA-Seq reads were first converted to a Sanger FASTQ format with FASTQ Groomer (version 1.0.4) and FASTQ Quality Trimmer (version 1.0.0) was applied to asses read quality scores [40,41]. Trimmed paired reads were mapped to reference genome with Tophat2 (version 0.6) tool [42], and Cufflinks (version 0.0.5) tools were used to assemble aligned reads into transcript/exon-isofoms [23]. The Cuffcompare (version 0.0.5) tool was applied to track transcripts across the time-points (4, 8 and 14 weeks of nodule age) and comparison of assembled transcripts to reference annotation. Finally, the Cuffdiff (version 0.0.5) tool was applied to find significant changes in transcription time points [23]. FPKM data (Fragments Per Kilobase of exon model per Million mapped fragments) generated were graphically represent data using the Multi-experiment viewer (MeV v4.8.1) software package [43]. The colour scale generated represents the transcription (FPKM) for each time point, normalized by subtracting the mean/median of three values from each individual value for each gene reduced by SD/RMS. * indicates significant change (p <0.05) in transcription between individual time points. Furthermore, FPKM data was compared to the data of [16] available online at SoySeq database [http://soybase.org/soyseq/]. Gene sequences were searched for any signal peptides with the online resource TargetP [http://www.cbs.dtu.dk/services/TargetP/] to determine any cellular localisation, results are summarised in Additional file 2. RNAseq data are available on Soybase (http://soybase.org/projects/SoyBase.A2014.01.php).

### Transcript quantification and RNA-Seq validation

Confirmation of transcription obtained from RNAseq data was carried out by quantitative real-time PCR (QPCR) after DNase I (1 U/μl) treatment of RNA and cDNA synthesis with the Thermo Scientific RevertAid First Strand cDNA Synthesis Kit (Qiagen, Germany). Reverse transcription was carried out in a 20 μl reaction volume with 1 μg RNA, 0.5 μg Oligo(dT)_18_ primer (100 μM) and 1 μl of RevertAid™ M-MuVL Reverse Transcriptase (200 U/μl). Reaction was carried out at 42°C for 60 min prior to inactivation at 70°C for 5 min. Primers for QPCR were designed with the IDT’s PrimerQuest Design Tool [http://eu.idtdna.com/PrimerQuest/Home/Index] and primer sets were applied at 300 nM (Additional file 4). The Bio-Rad CFX96-C1000 Thermal cycling was done with Touch Lightcycler with an initial 95°C for 10 min followed by cycling with 95°C for 15 seconds, 60°C for 30 seconds and 72°C for 30 seconds over 40 cycles. Specificity of PCR amplification was confirmed by melting curve analysis (75°C to 95°C) and sequencing of PCR amplicons. Amplicon specificity was screened by BLAST searches to detect any off-targets. Reverse transcriptase negative controls were used once for each RNA sample to detect any genomic DNA contamination. All reactions were setup in triplicates. The Bio-Rad CFX Manager v2.1 software was applied for data analysis and calculating C_q_. Any outliers were determined by Grubbs’s test and were removed from subsequent analysis [44,45]. Housekeeping genes applied for normalization were ribosomal protein 40S subunit S8 (40S) or elongation factor 1 beta (ELF1) [46] and SYBR Green I NTCs threshold of C_q_s 40 was used. Relative quantification and normalisation was done with the ΔΔCq method and transcript quantification was done twice to determine reproducibility. Each standard curve for each primer set was measured in triplicate and was checked for validity and primer pairs were only accepted if their standard curves had a slope between −3.3 and −3.8. Only R^2^ and PCR efficiencies between 90% and 110% (.90 ≤ Cq ≤1.1) was accepted.

### Phylogenetic analysis of cysteine proteases and cystatins

Full-length protein sequences for each of the cystatins and cysteine proteases were aligned and phylogenetic trees generated with the CLC Main Workbench v6.7.1. Neighbour Joining algorithm was applied with 100 Bootstrapping replicates. Model representative sequences for the different cystatin subfamilies identified by [20] were applied for phylogenetic analysis: Hv-CPI1 (CAA72790), Hv-CPI2 (CAG38123), Hv-CPI3 (CAG38124), Hv-CPI4 (CAG38130), Hv-CPI5 (CAG38126), Hv-CPI6 (CAG38127), Hv-CPI7 (CAG38131), Hv-CPI8 (CAG38129), Hv-CPI9 (CAG38125), Hv-CPI10 (CAG38128), Hv-CPI11 (CAG38132), Hv-CPI12 (CAG38133), Hv-CPI13 (CAG38134), as well as Monellin cystatin (At5g47550), Cystatin A (At2g40880), Cystatin B (At3g12490), Phytocystatin 2 (At2g31980) and a representative of the I25B cystatin from *Vigna unguiculata*. Out-group for the cystatin phylogenetic analysis consisted of papain.

Model representative sequences for the 8 different cysteine proteases subfamilies described by [21] were RD21A (At1g47128), RD21B (At5g43060), RD21C (At3g19390), RDL2 (At3g19400), XBCP3 (At1g09850), XCP1 (At4g35350), XCP1 (At1g20850), THI1 (At1g06260), SAG12 (At5g45890), RD19A (At4g39090), RD19B, (At2g21430), RD19C (At4g16190), AALP (At5g60360). ALP2 (At3g45310) and CTB3 (At4g1610) were also included in the phylogenetic trees to infer possible functional activity of the proteases. Out-group used for the C1 cysteine protease phylogenetic analysis was OCI (Os01g58890) and a further I25B cystatin from *Vigna unguiculata* (Q06445).

### Recombinant cystatin expression

Gene sequences for selected cystatins (Glyma04g10360, Glyma07g39590, Glyma08g11210 and Glyma13g27980 as well as each of the domains from Glyma14g04260, Glyma15g36180 and Glyma18g12240) were synthesized by GenScript. Sequences were synthesised with a 5’-BamHI and 3’-EcoRI restriction enzyme cut site for subsequent sub-cloning. Gene sequences of remaining cystatins (Glyma05g28250, Glyma13g04250, Glyma14g04250, Glyma20g08800) were isolated from cDNA preparations with gene specific primers (Additional file 5). Forward primers had a 5’-BamHI restriction enzyme site and reverse primers had a 3’-EcoRI restriction enzyme recognition sites for sub-cloning. Identified putative gene sequences were cloned into the plasmid pGEX-3X (Amersham Pharmacia Biotech, UK) as BamHI-EcoRI fragments and the *E. coli* strain BL21 (DE3) (Invitrogen, USA) was used for recombinant cystatin expression. All chemicals for bacteria culturing and the GenElute™ plasmid extraction kit for plasmid preparations were sourced from Sigma Aldrich (UK). All molecular biology enzymes, e.g. polymerases used for PCR isolation of gene sequences and enzymes used for cloning were sourced from Thermo Scientific (USA). Thermo Scientific GSH-agarose was applied during the protein purification procedure and Factor Xa used during the recombinant protein purification process (NEB, UK). Analysis of protein preparations throughout the recombinant protein expression process was done by SDS-PAGE [47] and protein quantification was carried out with a commercial protein determination assay [48].

### Determination of *Ki* values

Fluorogenic substrate Z-Phe-Arg-MCA (cathepsin L-like substrate from Sigma-Aldrich) was used at 10 μM final concentrations from a 400 μM stock dissolved in DMSO (Sigma-Aldrich, UK). Papain (Sigma; EC 3.4.22.2, UK), cathepsin-L (Sigma; EC 3.4.22.15, UK) and cathepsin-B (Sigma; EC 3.4.22.1, UK) were used as protease standards. Z-Phe-Arg-7-amino-4-methylcoumarin (Z-FR-AMC) and Z-Arg-Arg-7-amido-4-methylcoumarin (Z-RR-AMC) were applied as cysteine protease substrates to assay for cathepsin-L and cathepsin-B like activity. (Z-FR-AMC/Z-RR-AMC), cathepsin-F (Z-FR-AMC), cathepsin-H (Z-RR-AMC) and cathepsin-L (Z-FR-AMC) cysteine protease activity. Cysteine protease activity was determined and the *K*_*i*_ values for each of the different recombinant cystatins determined. Dissociation (inhibition) constants (*K*_*i*_) for the interaction between the different recombinant cystatins, with model cysteine proteases were determined according to [49]. Substrate hydrolysis progress curves were monitored as described by [50], and the linear equation was determined as described by [51]. Papain (pH 7.0), cathepsin L (pH 5.5) and cathepin B (pH 6.0) activity was measured in 50 mM sodium phosphate buffer, 4 mM EDTA and 8 mM L-cysteine at their respective enzyme pH optima and hydrolysis was at 25°C. Cysteine protease activities were determined with a Fluostar Galaxy fluorimeter (BMG, Germany), using a 360 nm excitation filter and a 450 nm emission filter. *K*_*m*_ values were 13.6 μM for papain, 2.0 μM for cathepsin B and 1.0 μM for cathepsin L [49]. The slope per sec (FU/sec) was calculated using the MARS Data Analysis Software v2.10 (BMG, Germany). E-64 (Sigma-Aldrich, UK) was applied as a broad spectrum inhibitor (positive control) for cysteine proteases at a concentration of 10nM [52]. Concentration of the model cystatin OCI was first tested to reduce proteolytic activity by 40-60% under assay conditions and an identical concentration was used to assay inhibitory potency of different soybean cystatins. The blank is represented by the slope/sec of buffer and substrate without enzymes, whereas the negative control is represented by the slope/sec of the uninhibited protease standards. All reactions were carried out in triplicate.

### Measurement of cystatin potency

Total plant protein extracts were applied as sources for cysteine protease activity in assays to measure cystatin potency. Extracts were prepared from soybean crown nodules corresponding to different time points (4, 8 and 14 weeks). Nodules were homogenised by crushing in liquid nitrogen and 50 mM sodium phosphate buffer, pH 6.0 was added in a 1:3 ration (50 mg : 150 μl; sample - buffer). Solution was incubated for 30 min on ice before centrifuging at 15000 g for 15 min at 4°C to remove any debris. Supernatant was removed, the total protein concentration determined, and a total of 100 ng protein was used per enzyme reaction. Protease activity measured was expressed as percentage relative to absence of inhibitor. ID_50_ for each cystatin was calculated as cystatin concentration required to achieve 50% inhibition of proteolytic activity. All reactions were carried out in triplicates.

### Statistical analysis

To determine significant transcription changes in the RNA-Seq data, a False Discovery Rate of 0.05 was used and significance in change was determined after the Benjamini-Hochberg correction for multiple-testing was applied. For generation of heat maps with the MeV software package, the Pearson’s correlation coefficient was used. A one-way ANOVA with Bonferroni post-tests was performed with GraphPad Prism Software version 5.00 for Windows (www.graphpad.com).

### Availability of supporting data

The data sets supporting the results of this article are available on Soybase, [BioProject: PRJNA261105; http://soybase.org/projects/SoyBase.A2014.01.php] or included in Additional files 1, 2, 3, 4 and 5.

## Additional files

Additional file 1:
**Cystatin sequences identified in soybean nodules by RNAseq analysis with similarity to oryzacystatin-I.** * indicates cystatins transcriptionally active in nodules. Additional file 2:
**The predicted signal peptide data generated by TargetP, include the Name, Length of protein, Final NN scores of final prediction (cTP, mTP, SP and other), Prediction of localization (Loc), Reliability class (RC), TPlen (Predicted presequence length), Chloroplast (C), Mitochondrion (M), Secretory pathway (S) and any other location (−).** The reliability classes are indicated as differences (diff) between the best second best prediction, expressed from high to low; 1: diff >0.800, 2: 0.800 > diff >0.600, 3: 0.600 > diff >0.400, 4: 0.400 > diff >0.200 and 5: 0.200 > diff. Additional file 3:
**Cysteine protease sequences identified in soybean nodules by RNAseq analysis with similarity to papain.** * indicates cysteine proteases transcriptionally active in nodules. Additional file 4:
**Primer sets to amplify of target transcripts.**
Additional file 5:
**Primer sets to isolate target cystatin gene sequences.**

## Abbreviations

FPKM: Fragments Per Kilobase of exon model per Million mapped fragments
PCD: Programmed cell death
